# Strategies for genetic inactivation of long noncoding RNAs in zebrafish

**DOI:** 10.1261/rna.069484.118

**Published:** 2019-08

**Authors:** Perrine Lavalou, Helene Eckert, Louise Damy, Florian Constanty, Sara Majello, Angelo Bitetti, Antoine Graindorge, Alena Shkumatava

**Affiliations:** Institut Curie, PSL Research University, CNRS UMR3215, INSERM U934, 75005 Paris, France

**Keywords:** CRISPR-Cas9, hypomorph, long noncoding RNAs, poly(A) signal, zebrafish

## Abstract

The number of annotated long noncoding RNAs (lncRNAs) continues to grow; however, their functional characterization in model organisms has been hampered by the lack of reliable genetic inactivation strategies. While partial or full deletions of lncRNA loci disrupt lncRNA expression, they do not permit the formal association of a phenotype with the encoded transcript. Here, we examined several alternative strategies for generating lncRNA null alleles in zebrafish and found that they often resulted in unpredicted changes to lncRNA expression. Removal of the transcription start sites (TSSs) of lncRNA genes resulted in hypomorphic mutants, due to the usage of either constitutive or tissue-specific alternative TSSs. Deletions of short, highly conserved lncRNA regions can also lead to overexpression of truncated transcripts. In contrast, knock-in of a polyadenylation signal enabled complete inactivation of *malat1*, the most abundant vertebrate lncRNA. In summary, lncRNA null alleles require extensive in vivo validation, and we propose insertion of transcription termination sequences as the most reliable approach to generate lncRNA-deficient zebrafish.

## INTRODUCTION

Thousands of lncRNAs have been identified in multiple vertebrate species (Necsulea et al. 2014; Hezroni et al. 2015), but their biological functions remain mostly unknown. To study lncRNAs in vivo, genetic mutants have been generated in model animals, primarily using a mouse model (Leighton et al. 1995; Marahrens et al. 1997; Ripoche et al. 1997; Sado et al. 2001; Sleutels et al. 2002; Bond et al. 2009; Eissmann et al. 2012; Nakagawa et al. 2012, 2014; Zhang et al. 2012; Grote et al. 2013; Li et al. 2013; Sauvageau et al. 2013; Han et al. 2014, 2018; Goff and Rinn 2015; Lai et al. 2015; Amândio et al. 2016; Anderson et al. 2016; Ip et al. 2016; Kotzin et al. 2016; Isoda et al. 2017; Kleaveland et al. 2018), but have also more recently been reported in zebrafish (Kok et al. 2015; Hosono et al. 2017; Bitetti et al. 2018; Goudarzi et al. 2019).

**FIGURE 1.**
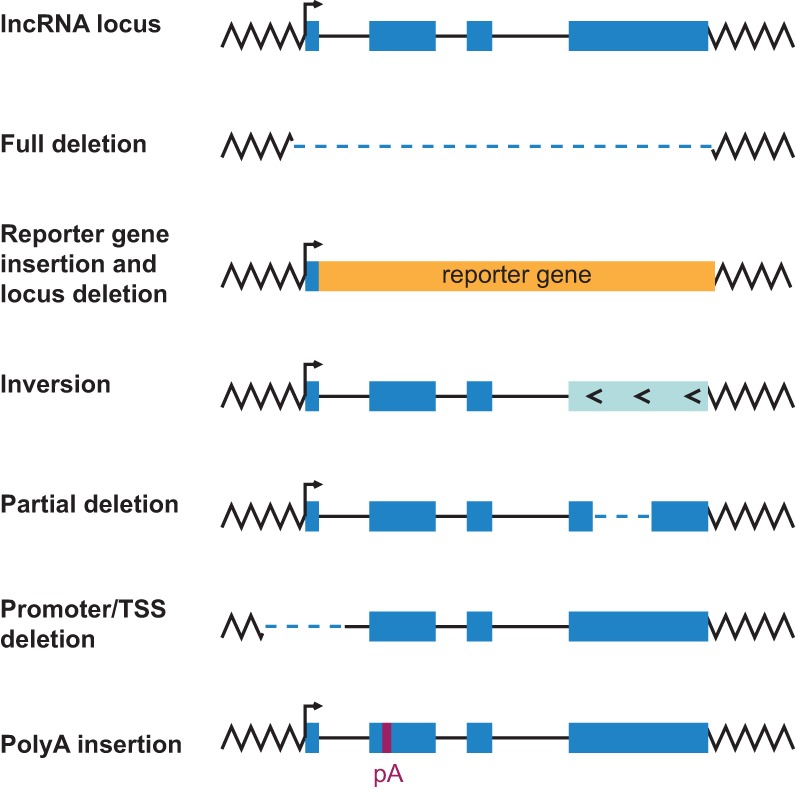
Strategies for genetic inactivation of lncRNAs in animals.

Genetic inactivation of lncRNAs is less straightforward than for coding genes, where deletion of an exon or a point mutation in the open reading frame (ORF) often leads to stop codons or frame-shift mutations and subsequent loss of function. Several complementary strategies have been implemented to achieve genetic loss of lncRNA function, including full or partial deletion of the lncRNA locus, deletion and subsequent replacement of the lncRNA locus by a reporter gene (Nakagawa et al. 2012; Sauvageau et al. 2013), deletion of the lncRNA transcription start site (TSS) and upstream regulatory regions (Fitzpatrick et al. 2002; Zhang et al. 2012) and sequence inversions (Fig. 1; Bitetti et al. 2018). Although commonly used, these lncRNA inactivation strategies have several caveats and limitations. Full deletions of lncRNA loci, which often span several kilobases, or lncRNA replacement by a reporter gene are invasive and might lead to phenotypes that are caused by removal of regulatory DNA motifs. Deletions of lncRNA TSS and upstream promoter regions may result in usage of alternative TSSs or cryptic promoters and/or impact the expression of neighboring genes. A less invasive and more accurate approach is to inactivate lncRNAs by integrating a premature polyadenylation [poly(A)] cassette. This strategy has been successfully implemented in several recent mouse lncRNA studies (Fig. 1; Bond et al. 2009; Grote et al. 2013; Anderson et al. 2016; Ballarino et al. 2018). Whereas lncRNA locus deletion and partial lncRNA gene inversion strategies have been applied in zebrafish to genetically inactivate lncRNAs (Kok et al. 2015; Hosono et al. 2017; Bitetti et al. 2018; Goudarzi et al. 2019), analyses of complementary lncRNA silencing approaches including the minimally invasive insertion of the poly(A) sequences have not yet been carried out.

Here, we examined the efficiency of several strategies for CRISPR-Cas9-mediated inactivation of lncRNAs in zebrafish. Careful evaluation of lncRNA zebrafish mutants demonstrated that caution is required when analyzing each individual mutant allele. When genetically manipulating lncRNA loci, we found that usage of constitutive or tissue-specific alternative TSSs, overexpression or destabilization of truncated lncRNA transcripts commonly take place in vivo, minimizing or confounding the effect of the intended genetic intervention. In contrast, using our minimally invasive knock-in of a premature polyadenylation signal into the *malat1* locus diminished *malat1* transcripts to undetectable levels, effectively establishing a *malat1* null allele in zebrafish.

## RESULTS

### Deletion of the conserved region of the lncRNA *cyrano* leads to overexpression of the truncated transcript

**FIGURE 2.**
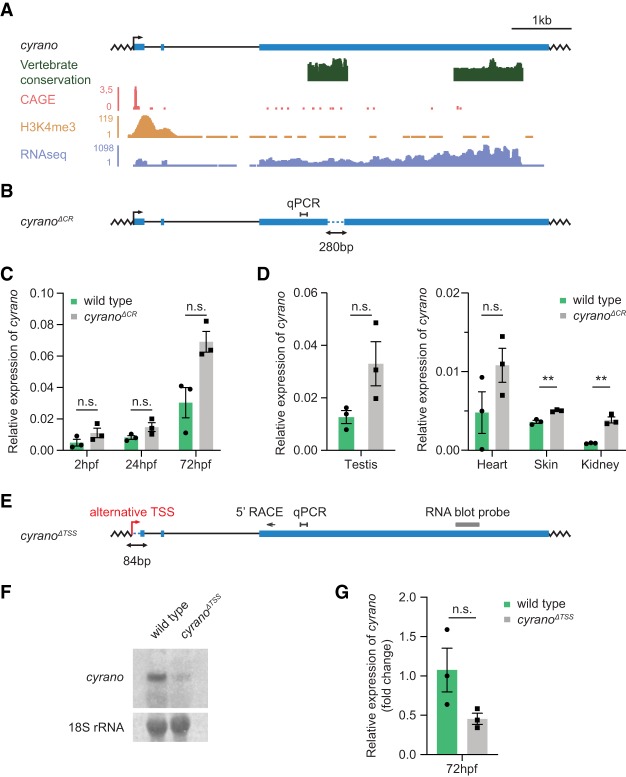
Genetic perturbations of the lncRNA *cyrano* in zebrafish result in overexpression and hypomorphic alleles. (*A*) Gene architecture of the lncRNA *cyrano*. Shown are the corresponding CAGE (Nepal et al. 2013; Haberle et al. 2014), H3K4me3 ChIP-Seq (Ulitsky et al. 2011), and RNA-seq tracks from wild-type (WT) zebrafish. Vertebrate conservation plots based on the eight-genome alignment indicate the location of conserved sequences. (*B*) The *cyrano*^*ΔCR*^ mutant allele showing the deletion of the most conserved region of the transcript (dotted, blue line) in zebrafish. Position of the qRT-PCR product is indicated. (*C*) *cyrano* expression in WT and homozygous *cyrano*^*ΔCR*^ embryos detected by qRT-PCR at 2 h postfertilization (hpf), 24 and 72 hpf. (*D*) *cyrano* expression across WT and homozygous *cyrano*^*ΔCR*^ adult tissues detected by qRT-PCR. (*E*) The *cyrano*^*ΔTSS*^ zebrafish allele showing deletion of the sequence around the TSS (dotted, blue line). Indicated are positions of the 5′ RACE primer, qPCR product, RNA blot probe and alternative TSS. (*F*) *cyrano* expression in 72 hpf WT and homozygous *cyrano*^*ΔTSS*^ embryos detected by an RNA blot. 18S rRNA was used as a reference gene. (*G*) *cyrano* expression in 72 hpf WT and homozygous *cyrano*^*ΔTSS*^ embryos detected by qRT-PCR. *eef1α1l1* was used as a reference gene in all qRT-PCR experiments. Each dot represents an individual biological replicate. Data are presented as mean ± S.E.M.; (*) *P* < 0.05, n.s., not significant, unpaired *t*-tests.

A small fraction of zebrafish lncRNAs are conserved in mammals, representing a promising set of candidates for functional interrogation (Ulitsky et al. 2011; Hezroni et al. 2015). The conserved regions of lncRNAs are usually relatively short, ranging between 50–300 nucleotides (nt) (Ulitsky et al. 2011; Hezroni et al. 2015) and can be efficiently targeted for CRISPR-Cas9-mediated deletions in zebrafish, offering a minimally invasive strategy for functional inactivation (Fig. 1). To examine the effect of this strategy on lncRNA expression, we chose the deeply conserved lncRNA *cyrano* (Ulitsky et al. 2011) for genetic interrogations in zebrafish. We generated a ∼280 base pair (bp) deletion of the most conserved region of the 5.5 kb sequence, hereafter referred as *cyrano*^*ΔCR*^ (Fig. 2A,B; Ulitsky et al. 2011). Interestingly, we detected elevated levels of the residual truncated transcript in homozygous *cyrano*^*ΔCR*^ zebrafish embryos and across *cyrano*^*ΔCR*^ adult tissues apart from the brain (Fig. 2C,D; Supplemental Fig. 1A). These results suggest that removal of a relatively small region of a lncRNA may have an unexpected effect on the transcript levels, potentially leading to its unintended overexpression.

### TSS deletion of the *cyrano* locus results in hypomorphic zebrafish mutants

Next, we tested if deleting the sequences surrounding and containing lncRNA TSS elements is a reliable alternative strategy for zebrafish lncRNA genetic inactivation. To this end, we generated a minimally invasive *cyrano*^*ΔTSS*^ mutant allele by removing sequences containing the *cyrano* TSS (0 to +84) (Fig. 2E). Although *cyrano* transcript levels were reduced in *cyrano*^*ΔTSS*^ fish, the transcript was still robustly detectable by RNA blot analysis and qRT-PCR, resulting in a hypomorphic *cyrano*^*ΔTSS*^ mutant (Fig. 2F,G). The 5′ RACE (rapid amplification of cDNA ends) analysis demonstrated that in the absence of the two main TSSs usually used in WT animals, an alternative upstream TSS maintains *cyrano* expression in *cyrano*^*ΔTSS*^ mutant zebrafish (Supplemental Fig. 1B–D).

Notably, neither the *cyrano*^*ΔCR*^ mutant, with removal of the highly conserved miR-7 site (Ulitsky et al. 2011), nor the *cyrano*^*ΔTSS*^ mutant fish exhibited obvious morphological defects. This observation is consistent with recent zebrafish and mouse studies (Kleaveland et al. 2018; Goudarzi et al. 2019) and is in contrast to previous studies that used a morpholino-based knockdown approach to inactivate *cyrano* (Ulitsky et al. 2011; Sarangdhar et al. 2018).

### lncRNA TSS removal leads to tissue-specific alternative TSS usage, maintaining lncRNA expression

**FIGURE 3.**
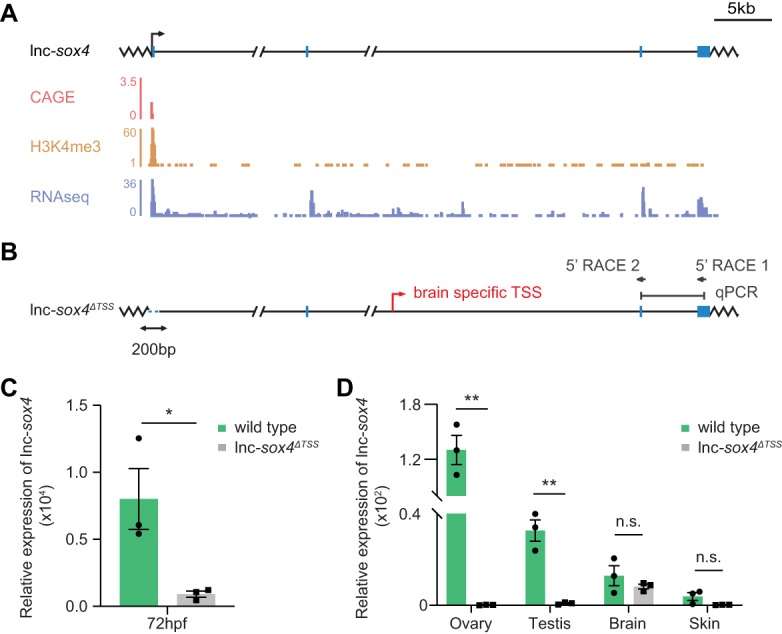
Presence of a tissue-specific alternative TSS leads to a brain-specific rescue of lnc-*sox4a* expression. (*A*) The lnc-*sox4a* locus in zebrafish (chr19:29,161,676-29,270,573). Shown are the corresponding CAGE (Nepal et al. 2013; Haberle et al. 2014), H3K4me3 ChIP-Seq (Ulitsky et al. 2011), and RNA-seq tracks from WT zebrafish. (*B*) The lnc-*sox4a*^*ΔTSS*^ mutant allele showing deletion of the sequence around the TSS (dotted, blue line). Indicated are positions of the 5′ RACE primers, qPCR primers, and alternative TSS. (*C*) lnc-*sox4a* expression in 72 h postfertilization (hpf) WT and homozygous lnc-*sox4a*^*ΔTSS*^ embryos detected by qRT-PCR. (*D*) lnc-*sox4a* expression across adult WT and homozygous lnc-*sox4a*^*ΔTSS*^ zebrafish tissues detected by qRT-PCR. *eef1α1l1* was used as a reference gene in all qRT-PCR experiments. Each dot represents an individual biological replicate. Data are presented as mean ± S.E.M.; (*) *P* < 0.05, (**) *P* < 0.01, n.s., not significant, unpaired *t*-tests.

To test if the usage of alternative TSSs is a prevalent cellular mechanism to maintain lncRNA gene expression, we examined the effect of TSS deletions on additional lncRNAs in zebrafish. We generated a lnc*-sox4a*^*ΔTSS*^ mutant allele by removing ∼200 bp surrounding the lnc-*sox4a* TSS (−43 to +157) (Fig. 3A,B). lnc-*sox4a* (chr19:29,161,676-29,270,573; Zv9/danRer7) (Ulitsky et al. 2011) is highly expressed in the zebrafish ovary and was successfully abolished in lnc*-sox4a*^*ΔTSS*^ embryos and across lnc*-sox4a*^*ΔTSS*^ adult tissues (Fig. 3C,D). However, lnc-*sox4a* was robustly expressed in the adult lnc*-sox4a*^*ΔTSS*^ brain at levels comparable to WT (Fig. 3D). The 5′ RACE analysis confirmed that a tissue-specific alternative TSS, located in an intron 70 kb downstream from the main TSS (Fig. 3B; Supplemental Fig. 2A,B), was used only in the lnc*-sox4a*^*ΔTSS*^ animals and maintained lncRNA expression specifically in the adult brain (Fig. 3D). While homozygous lnc*-sox4a*^*ΔTSS*^ fish were viable and fertile, our alternative strategy to eliminate lnc-*sox4a* expression by deleting the last exon failed to generate homozygous fish (Supplemental Fig. 2C,D).

**FIGURE 4.**
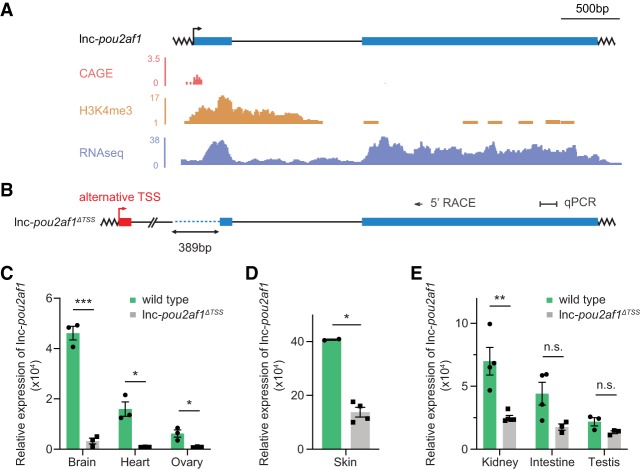
Usage of tissue-specific alternative TSSs maintains lnc-*pou2af1* expression in a subset of adult tissues. (*A*) The lnc-*pou2af1* locus in zebrafish. Shown are the corresponding CAGE (Nepal et al. 2013; Haberle et al. 2014), H3K4me3 ChIP-Seq (Ulitsky et al. 2011), and RNA-seq tracks from WT zebrafish. (*B*) The lnc-*pou2af1*^*ΔTSS*^ mutant allele showing deletion of the sequence around the TSS (dotted, blue line). Indicated are positions of the 5′ RACE and qPCR primers and alternative TSS. Red box represents a new exon generated from the alternative TSS. (*C*–*E*) lnc-*pou2af1* expression across a subset of adult WT and homozygous lnc-*pou2af1*^*ΔTSS*^ zebrafish tissues detected by qRT-PCR. *eef1α1l1* was used as a reference gene in all qRT-PCR experiments. Each dot represents an individual biological replicate. Data are presented as mean ± S.E.M.; (*) *P* < 0.05, (**) *P* < 0.01, (***) *P* < 0.001, n.s., not significant, unpaired *t*-tests.

We generated an additional lncRNA mutant by removing ∼390 bp surrounding the lnc-*pou2af1* TSS (−74 to +315) (Fig. 4A,B). Similar to the lnc*-sox4a*^*ΔTSS*^ allele, the level of lnc*-pou2af1* (chr15:16770170-16773603; Zv9/danRer7) was abolished in lnc*-pou2af1*^*ΔTSS*^ embryos and in a subset of tested lnc*-pou2af1*^*ΔTSS*^ adult tissues (Fig. 4C; Supplemental Fig. 3A). However, in skin, kidney, intestine and testis, expression of lnc*-pou2af1* was robustly detected in lnc*-pou2af1*^*ΔTSS*^ fish (Fig. 4D,E). The 5′ RACE analysis showed that several alternative TSSs, located ∼1 kb upstream of the main TSS, were used in the lnc*-pou2af1*^*ΔTSS*^ animals in a tissue-specific manner (Fig. 4B; Supplemental Fig. 3B,C). Expression of lnc*-pou2af1* from alternative TSSs generated new tissue-specific lncRNA exons at the 5′ of the transcript (Fig. 4B; Supplemental Fig. 3C,D).

Together, our data showed that in the absence of the main TSS, alternative TSSs can be used in a tissue-specific manner, generating hypomorphic mutants, and minimizing the effect of the intended gene inactivation.

### Insertion of a polyadenylation signal resulted in a *malat1* null allele in zebrafish

**FIGURE 5.**
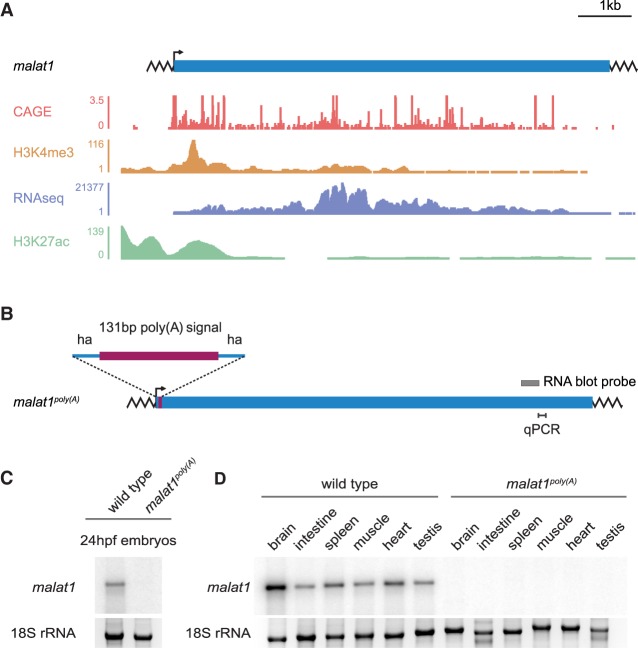
Effective inactivation of the lncRNA *malat1* in zebrafish by insertion of a premature polyadenylation signal. (*A*) The *malat1* locus in zebrafish. Shown are the corresponding CAGE (Nepal et al. 2013; Haberle et al. 2014), H3K4me3 ChIP-Seq (Ulitsky et al. 2011), RNA-seq, and H3K27ac ChIP-Seq (Pérez-Rico et al. 2017) tracks from WT zebrafish. (*B*) Generation of the *malat1*^*poly(A)*^ allele by targeted knock-in of the poly(A) signal. The hybridization site of the RNA blot probe is indicated as a gray box. ha, homology arms. (C) *malat1* expression in WT and homozygous *malat1*^*poly(A)*^ embryos detected by an RNA blot. 18S rRNA was used as a loading reference. hpf, hours postfertilization. (*D*) *malat1* expression across WT and homozygous *malat1*^*poly(A)*^ adult zebrafish tissues detected by RNA blot. 18S rRNA was used as a loading reference.

Given the evidence that usage of alternative TSSs may be a common cellular mechanism to confer lncRNA expression, we tested if knock-in of a poly(A) signal into a lncRNA locus can be applied in zebrafish as a minimally invasive alternative to generate lncRNA null alleles. This approach has been successfully used to inactivate lncRNAs in mice (Grote et al. 2013; Anderson et al. 2016; Isoda et al. 2017; Ballarino et al. 2018). The *malat1* locus produces one of the most abundant lncRNAs in vertebrate genomes (Ulitsky et al. 2011; Hezroni et al. 2015). Because *malat1* is a mono-exonic lncRNA of ∼7.5 kb and its locus contains multiple TSSs and clustered enhancers forming a so-called super-enhancer (Pérez-Rico et al. 2017), any deletion strategy of the locus, including TSS removal, has a strong potential to affect *cis* regulatory elements (Fig. 5A). Therefore, we applied our improved protocol for the efficient targeted knock-in to insert a 131 bp SV40 poly(A) signal into the *malat1* locus in zebrafish (Fig. 5B; see Materials and Methods; Supplemental Fig. 4A). The targeted knock-in of the poly(A) sequence completely abolished *malat1* expression in zebrafish embryos and in all examined adult tissues (Fig. 5C,D; Supplemental Fig. 4B,C). Despite effective inactivation of *malat1*, *malat1*^*poly(A)*^ zebrafish were viable and fertile and displayed no obvious morphological defects. The lack of overall morphological abnormalities is consistent with previously reported *Malat1*^−/−^ mice (Eissmann et al. 2012; Nakagawa et al. 2012; Zhang et al. 2012) and is in contrast to morpholino-based *malat1* inactivation in zebrafish (Wu et al. 2018). Taken together, compared to lncRNA deletion strategies, poly(A) signal insertion was the most efficient and least invasive approach in zebrafish.

## DISCUSSION

The identification of lncRNAs in model vertebrates, their comparative genomics analyses and recent progress in genome editing technologies has led to the generation of multiple mutant lncRNA alleles. Because common strategies for genetic inactivation of lncRNAs often do not allow distinguishing between functions mediated by the lncRNA transcript and those mediated by overlapping DNA regulatory motifs, the generation and interpretation of lncRNA null alleles can be challenging. Here, we compared zebrafish lncRNA mutant alleles generated using several alternative and commonly applied CRISPR-Cas9 strategies for lncRNA inactivation.

We demonstrated that relatively small deletions of conserved regions of lncRNAs, which represent attractive target sequences to eliminate or diminish lncRNA functions (Bitetti et al. 2018; Kleaveland et al. 2018), might result in unexpected changes in lncRNA levels, such as overexpression of the remaining transcript, as demonstrated for *cyrano*. One possibility is that deletion of the conserved region of *cyrano*, which removed a highly conserved and extensively paired site to miR-7 (Ulitsky et al. 2011), stabilized the *cyrano* transcript in zebrafish. Alternatively, deletion of this region of *cyrano* in zebrafish might have caused transcriptional up-regulation. For example, if deletion of this region abrogated *cyrano* function, cells might have boosted transcription of the locus in an attempt to restore *cyrano* activity. Deletion of the conserved region of mouse *cyrano* does not lead to increased lncRNA levels (Kleaveland et al. 2018), which suggests that *cyrano* regulation has diverged between fish and mammals. A better understanding of *cyrano* regulation and function will help identify the source of this ectopic effect on the remaining lncRNA transcript observed in fish and how this effect might complicate interpretation of the deletion results.

Moreover, we showed that the removal of TSS and upstream regulatory regions, a commonly used approach considered to be straightforward to interpret, can result in the presence of either constitutive or tissue-specific alternative TSSs that preclude efficient inactivation of lncRNAs and result in hypomorph mutant animals. Although not shown in this study, usage of temporal-specific alternative TSSs might also contribute to the maintenance of lncRNA expression at specific developmental stages, complicating the analysis and interpretation of TSS mutant alleles in animal models. Interestingly, a recent study reported that a 326 bp deletion removing *cyrano's* TSS leads to loss of the lncRNA expression (Goudarzi et al. 2019). The difference observed between the *cyrano*^*ΔTSS*^ alleles may be a consequence of the larger deletion used by Goudarzi et al. potentially leading to a more effective down-regulation of *cyrano*. In addition, the choice of the lncRNA detection method as well as the developmental timing of detection are important. Our data show that in TSS deletion alleles, lncRNA expression is often abolished at early embryonic stages and robustly reestablished later during development by tissue-specific alternative TSSs. These collective observations underscore the necessity to carefully validate TSS deletion alleles.

Importantly, our improved protocol for efficient targeted knock-in in zebrafish enabled examination of the effect of a poly(A) signal insertion into the most abundant and enhancer-dense lncRNA locus. We demonstrate that this minimally invasive genome editing strategy, previously shown to be successful for lncRNA inactivation in mice (Grote et al. 2013; Anderson et al. 2016; Isoda et al. 2017; Ballarino et al. 2018), is a highly effective strategy in zebrafish. Given the ease of our knock-in approach, which combines the use of a single-strand oligo as a template for homologous recombination and inhibition of nonhomologous end joining, we anticipate that the insertion of a poly(A) sequence will become a widespread strategy for generating lncRNA mutant alleles in zebrafish. Furthermore, the knock-in strategy can be used for genetic tagging of lncRNAs with self-cleaving ribozymes, which has been demonstrated to perturb lncRNA expression in mouse embryonic stem cells (Tuck et al. 2018) but has not been tested yet in model organisms.

Taken together, evaluation of several independent lncRNA mutant alleles in zebrafish indicates that a combination of complementary lncRNA inactivation approaches and their careful analyses are required for robust and accurate lncRNA functional interrogation.

## MATERIALS AND METHODS

### Generation of lncRNA mutant alleles in zebrafish

All lncRNA mutant alleles were generated using CRISPR/Cas9-mediated genome editing. To generate lnc-*sox4a*^*ΔTSS*^, lnc-*pou2af1*^*ΔTSS*^, *cyrano*^*ΔTSS*^, and *cyrano*^*ΔCR*^ alleles, two sgRNAs (9 ng each, Supplemental Table 1) and 150 ng in vitro transcribed *Cas9* mRNA were coinjected into the one-cell stage AB zebrafish embryos (Hwang et al. 2013). To generate lnc-*sox4a*^*Δ3′exon*^ allele, two sgRNAs (100 ng each, Supplemental Table 1) and Cas9 protein (50 ng/µL, a gift of the Concordet Lab, Muséum d'Histoire Naturelle, Paris) were coinjected into the one-cell stage AB zebrafish embryos (Hwang et al. 2013). sgRNAs and *Cas9* mRNA were generated as described previously (Hwang et al. 2013), using the codon-optimized plasmid JDS246 for the *Cas9* mRNA synthesis (Addgene #43861), purified with RNeasyMini Kit (Qiagen). Genomic DNA was extracted as described previously (Bitetti et al. 2018) and used for genotyping by PCR, DNA sequencing and mapping of genetic amplification product. The genotyping primers are listed in Supplemental Table 2.

All zebrafish were bred and maintained at Institut Curie, Paris. Animal care and use for this study were performed in accordance with the recommendations of the European Community (2010/63/UE) for the care and use of laboratory animals. Experimental procedures were specifically approved by the ethics committee of Institut Curie CEEA-IC #118 (project CEEA-IC 2017-017) in compliance with the international guidelines. Zebrafish were staged using standard procedures (Kimmel et al. 1995).

### Generation of the *malat1*^*poly(A)*^ allele by CRISPR/Cas9-mediated homologous recombination in zebrafish

The CRISPR/Cas9-mediated knock-in protocol was optimized as described in Supplemental Figure 4A. Zebrafish *malat1*^*poly(A)*^ mutant was generated by insertion of a single SV40 poly(A) signal (131 bp) into the *malat1* locus. Briefly, one-cell stage embryos were injected with a single guide RNA (100 ng, Supplemental Table 1), Cas9 protein (50 ng/µL, a gift of the Concordet laboratory, Muséum d'Histoire Naturelle, Paris), a morpholino against *xrcc4* to suppress NHEJ (nonhomologous end joining) (3 ng/µL, Gene Tools LLC, Supplemental Table 1), and a 191 nt single-strand DNA oligo with 30 bp homology arms flanking both sides of the SV40 poly(A) sequence (200 ng, designed and manufactured by Ultramer IDT, Supplemental Table 1). Genomic DNA was extracted as described previously (Bitetti et al. 2018), and poly(A) insertion was detected by PCR using primers listed in Supplemental Table 2, DNA sequencing and mapping of genetic amplification product.

### qRT-PCR

Total RNA was isolated from zebrafish embryos and adult tissues by TRIzol extraction (Invitrogen) followed by DNase treatment (TURBO DNA-free Ambion). For individual replicates, RNA isolated from 30–100 embryos or tissues from one to six adult fish was used. cDNA was produced with SuperScript IV reverse transcriptase (Invitrogen) and amplified with PowerUp SYBR Green PCR Master Mix (ThermoFisher Scientific) using primers listed in Supplemental Table 3. For each biological replicate, qRT-PCRs were performed in technical triplicate. The *eef1α1l1* (*eukaryotic translation elongation factor 1 alpha 1, like 1*) was used as a reference gene (McCurley and Callard 2008).

### RNA blots

Total RNA was isolated using TRIzol (Invitrogen), separated on 1% agarose gels containing 0.8% formaldehyde, and transferred to nylon membrane (Nytran SPC, GE Healthcare) by capillary action. Blots were hybridized with α-UTP ^32^P-labeled RNA probes at 68°C in ULTRAhyb buffer (Ambion) as recommended by the manufacturer. RNA probe template was amplified from zebrafish brain cDNA by PCR using the primers listed in Supplemental Table 3 (the sequence of the T7 promoter is underlined) and in vitro transcribed (RNA Maxiscript, Ambion) in the presence of α-UTP^32^P. For each replicate, RNA isolated from 30–100 embryos or tissues from three to six adult fish was used. The gel blots and hybridizations in Figure 5C were performed in biological triplicates. The hybridizations in Figures 2F and 5D were performed once.

### RNA ligase-mediated and oligo-capping rapid amplification of cDNA ends (5′ RACE)

TSS usage was determined by rapid amplification of cDNA ends (RACE) according to manufacturer's instruction (GeneRacer kit, Life Technology). Gene specific primers listed in Supplemental Table 3 were used to amplify lncRNA 5′ RACE products through PCR and nested PCR, subcloned into the PCR BLUNT II TOPO vector (Invitrogen), and transformed in the NEB TOP-10 cells. A minimum of 12 colonies were sequenced, and the sequences were aligned to the corresponding lncRNA genomic locus.

## SUPPLEMENTAL MATERIAL

Supplemental material is available for this article.

## Supplementary Material

Supplemental Material
