# Supplementary material for: Strategies for genetic inactivation of long noncoding RNAs in zebrafish
Source: RNA. 2019 Aug;25(8):897–904. doi: 10.1261/rna.069484.118 (PMC6633201; doi:10.1261/rna.069484.118)
Supplement: Supplemental Material [file supp_25_8_897__index.html]

Strategies for Genetic Inactivation of Long Noncoding RNAs in Zebrafish — Strategies for genetic inactivation of long noncoding RNAs in zebrafish — Supplemental Material 

# Strategies for genetic inactivation of long noncoding RNAs in zebrafish

## Supplemental Material

- Supplemental\_Figure\_1.ai
- Supplemental\_Figure\_2.ai
- Supplemental\_Figure\_3.ai
- Supplemental\_Figure\_4.ai
- Supplemental\_Figure\_Legends.docx
- Supplemental\_Tables.docx
