# Supplementary figures and images for: Strategies for genetic inactivation of long noncoding RNAs in zebrafish
Source: RNA. 2019 Aug;25(8):897–904. doi: 10.1261/rna.069484.118 (PMC6633201; doi:10.1261/rna.069484.118)

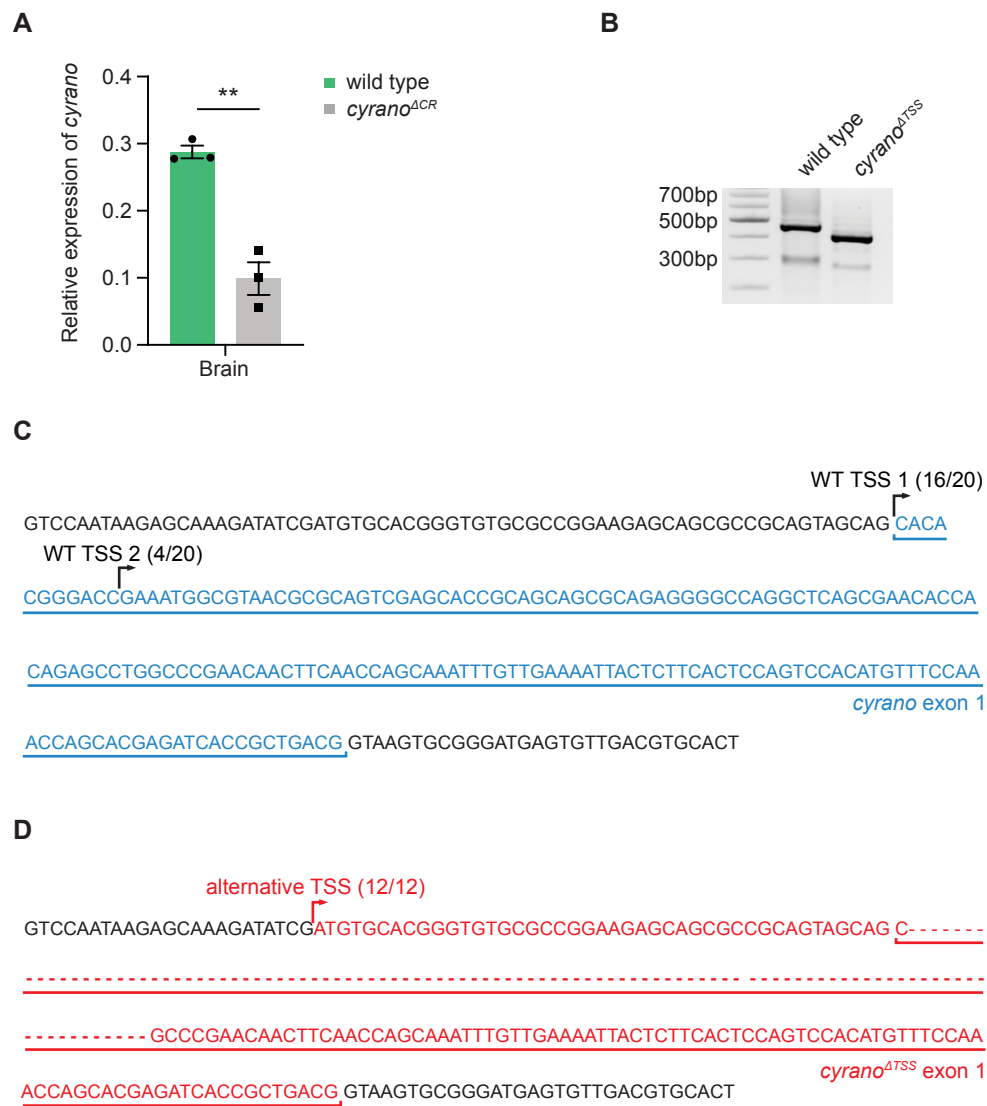

Supplement: Supplemental Material [file supp_069484.118_Supplemental_Figure_1.ps]

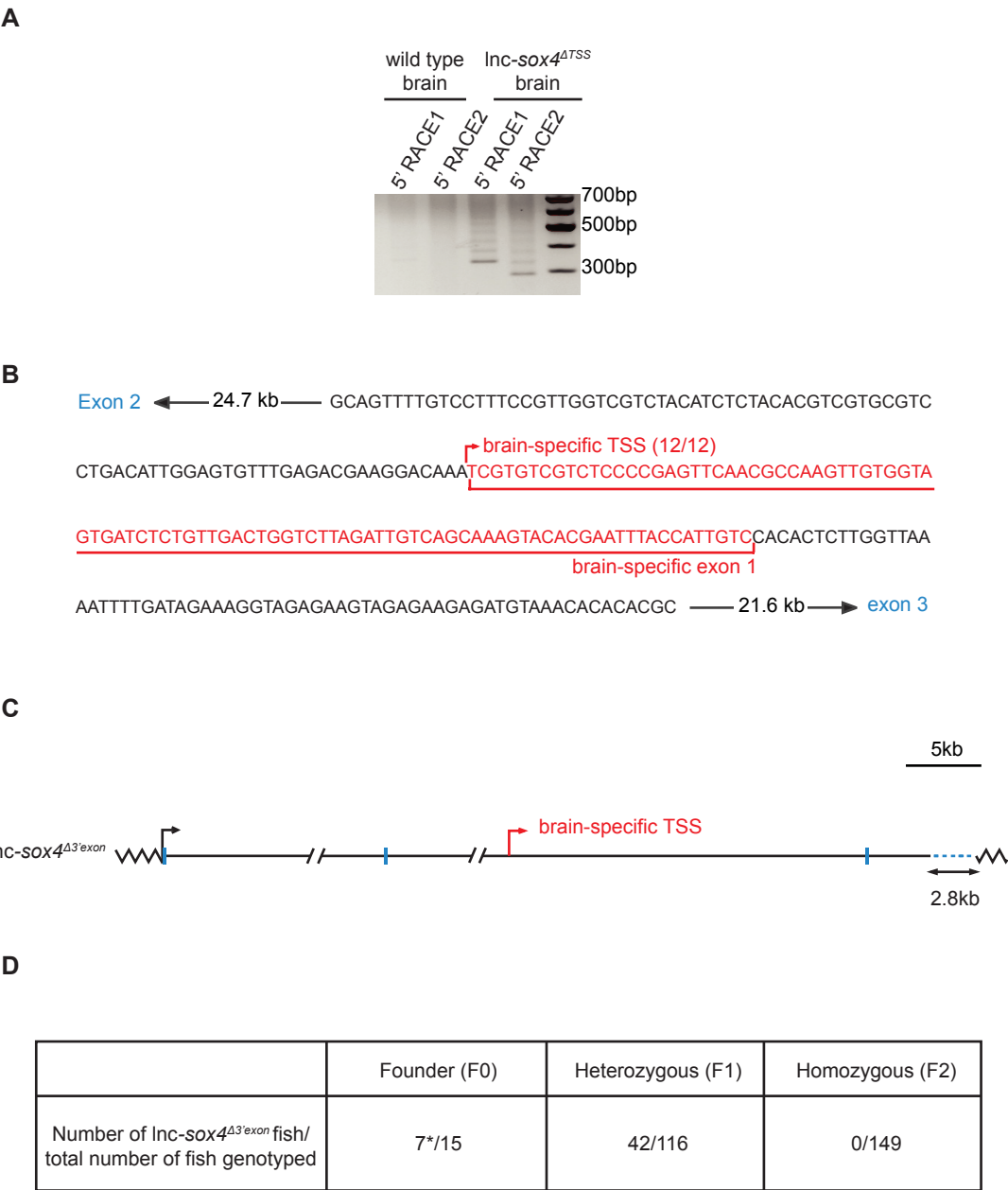

\* Germ line transmission rate ranging from 2/24 to 14/24

Supplement: Supplemental Material [file supp_069484.118_Supplemental_Figure_2.ps]

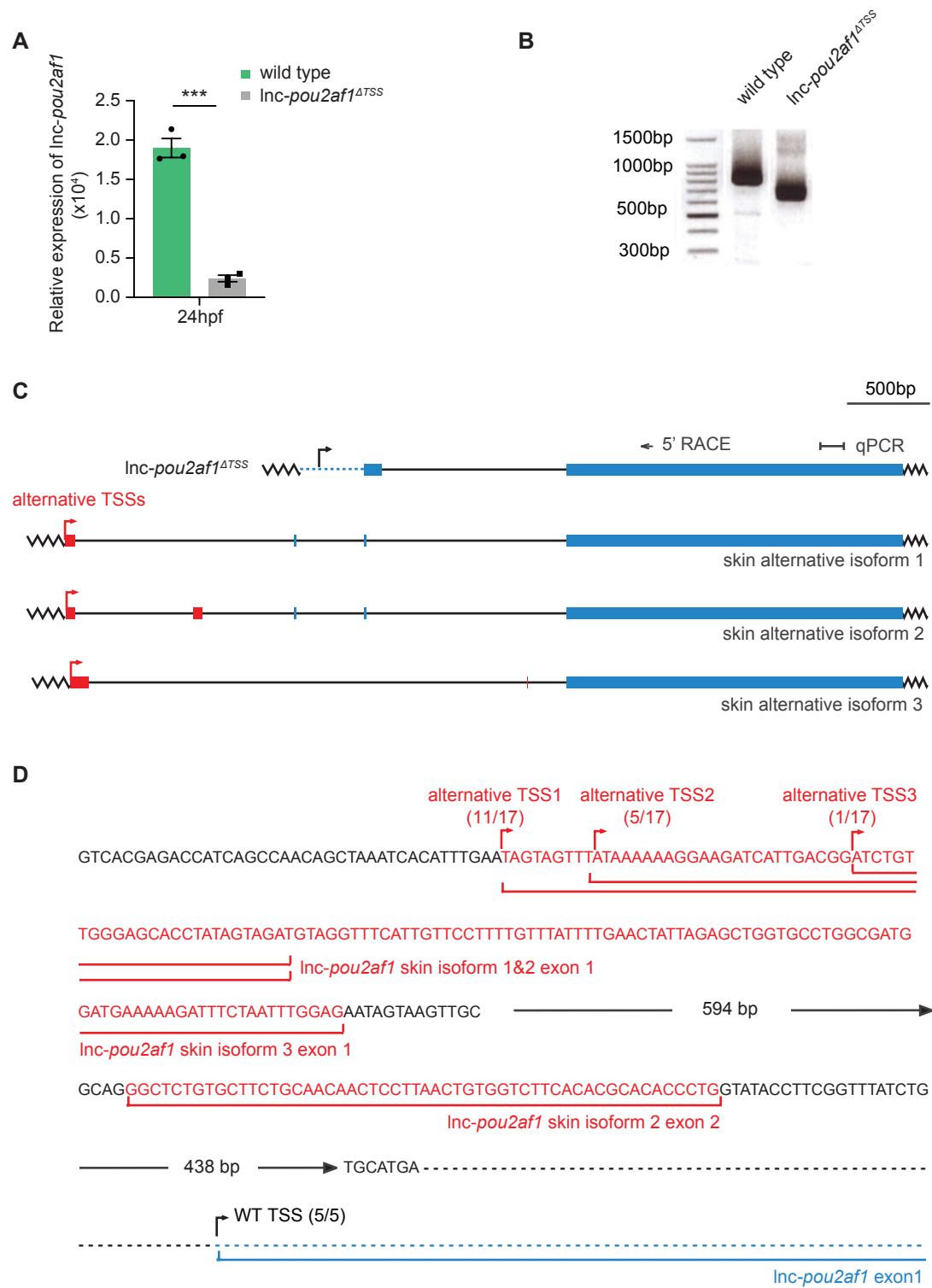

Supplement: Supplemental Material [file supp_069484.118_Supplemental_Figure_3.ps]

**A**

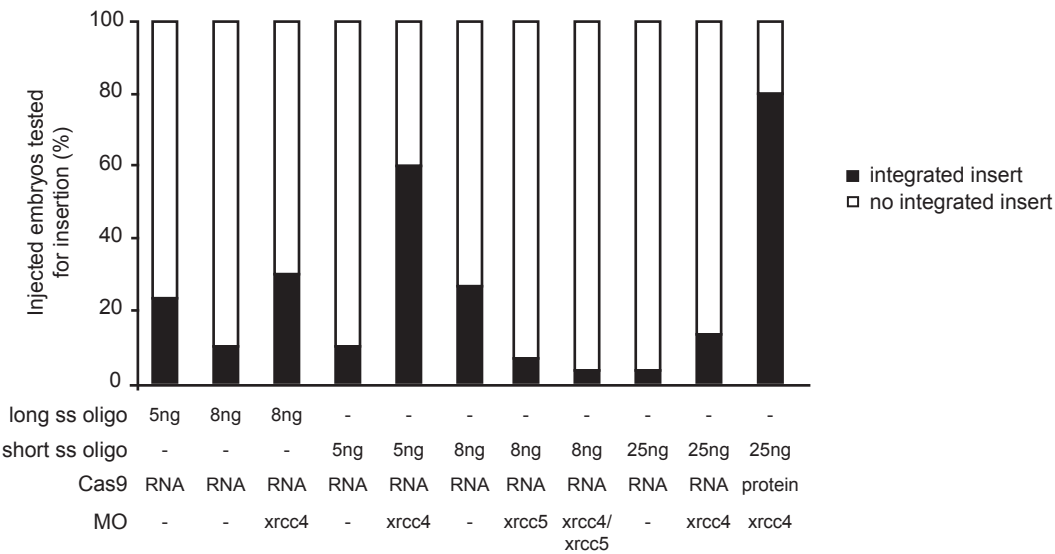

**B**

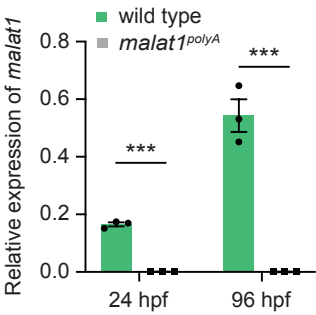

**C**

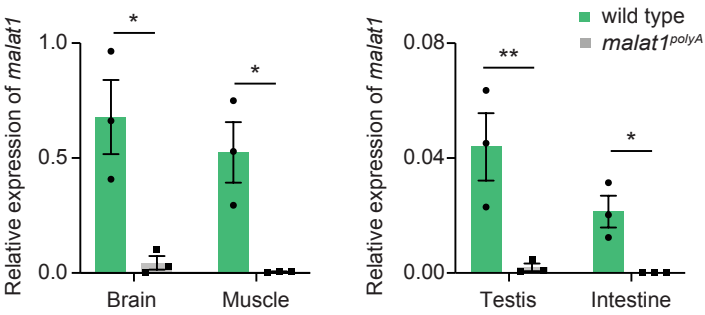

Supplement: Supplemental Material [file supp_069484.118_Supplemental_Figure_4.ps]
