# Supplementary material for: Strategies for genetic inactivation of long noncoding RNAs in zebrafish
Source: RNA. 2019 Aug;25(8):897–904. doi: 10.1261/rna.069484.118 (PMC6633201; doi:10.1261/rna.069484.118)
Supplement: Supplemental Material [file supp_069484.118_Supplemental_Figure_Legends.docx]

**Supplementary Fig. 1**

(A) *cyrano* expression in wild type and homozygous *cyrano^ΔCR^* adult brain detected by qRT-PCR. *eef1α1l1* was used as a reference gene in all qRT-PCR experiments. Each dot represents an individual biological replicate. Data are presented as mean ± s.e.m.; ***P* < 0.01, n.s., not significant, unpaired *t* tests. (B) DNA fragments amplified by 5’ RACE in 72 hpf wild type and homozygous *cyrano^ΔTSS^* embryos. (C) The sequence of the *cyrano* wild type locus in zebrafish. The nucleotides defining the borders of the first exon of *cyrano* are shown in blue and underlined. Positions of two main TSSs (WT TSS1 and WT TSS2) are indicated with arrows. The numbers in brackets show the frequency of TSS usage in wild type animals. (D) The sequence of the zebrafish *cyrano^ΔTSS^* allele. The nucleotides defining the borders of the first exon of *cyrano* are shown in red and underlined. The deletion is shown as a dotted line. The position of the alternative TSS is indicated with a red arrow. The numbers in the brackets show the frequency of the alternative TSS usage in *cyrano^ΔTSS^* embryos.

**Supplementary Fig. 2**

(A) DNA fragments amplified by 5’ RACE in the wild type and homozygous lnc-*sox4a^ΔTSS^* brain. (B) The sequence of the zebrafish lnc-*sox4a^ΔTSS^* allele. The nucleotides defining the borders of the tissue-specific exon expressed in the lnc-*sox4a^ΔTSS^* brain are shown in blue and underlined. The position of the alternative TSS is indicated with a red arrow. The intron sequences are indicated in black. (C) The lnc-*sox4a^Δ3’exon^* mutant allele showing deletion of the sequence at the 3’end of the gene (dotted, blue line). (D) Number of identified lnc-*sox4a^Δ3’exon^* fish.

**Supplementary Fig. 3**

(A) lnc-*pou2af1* expression in 24 hpf wild type and homozygous lnc-*pou2af1^ΔTSS^* embryos detected by qRT-PCR. *eef1α1* was used as a reference gene. Each dot represents an individual biological replicate. Data are presented as mean ± s.e.m.; ****P* < 0.001, n.s., not significant, unpaired *t* tests. (B) DNA fragments amplified by 5’ RACE in wild type and homozygous lnc-*pou2af1^ΔTSS^* skin. (C) The lnc-*pou2af1^ΔTSS^* mutant allele is shown on the top. Deletion of the sequence is shown as a dotted, blue line. lnc-*pou2af1* skin-specific expression from alternative TSSs (red arrows) results in multiple alternative isoforms. (D) The sequence of the zebrafish lnc-*pou2af1^ΔTSS^* allele. The nucleotides defining the borders of the tissue-specific exon expressed in the lnc-*pou2af1^ΔTSS^* skin are shown in red and underlined. The positions of the alternative TSSs are indicated with red arrows, intron sequences are indicated in black and deletion is shown as a dotted line.

**Supplementary Fig. 4**

(A) Optimization of the knock-in protocol in zebrafish. One-cell stage zebrafish embryos were co-injected with CRISPR/Cas9, single strand oligonucleotides (oligos) and anti-sense morpholinos (MO) targeting the DNA repair pathway. Cas9 was injected either as mRNA or protein. Different concentrations of single strand (ss) oligos with either 30bp (short ss oligo) or 60bp (long ss oligo) homology arms were tested. To suppress non-homologous end joining (NHEJ) repair, translation of either *xrcc4* (DNA ligase 4) or *xrcc5* (Ku80) was inhibited by their respective morpholinos. All conditions were tested for the *lnc-klf7b* locus (chr9:28,399,359-28,403,803). To evaluate the knock-in efficiency, PCR was performed on single embryos at 48 hpf. 30 embryos per condition were tested.

(B) *malat1* expression in the wild type and homozygous *malat1^ΔpolyA^* embryos detected by qRT-PCR. (C) *malat1* expression across wild type and homozygous *malat1^polyA^* adult zebrafish tissues detected by qRT-PCR. *eef1α1l1* was used as a reference gene in all qRT-PCR experiments. Each dot represents an individual biological replicate. Data are presented as mean ± s.e.m.; ***P* < 0.01, ****P* < 0.001, n.s., not significant, unpaired *t* tests.
