# Supplementary material for: Strategies for genetic inactivation of long noncoding RNAs in zebrafish
Source: RNA. 2019 Aug;25(8):897–904. doi: 10.1261/rna.069484.118 (PMC6633201; doi:10.1261/rna.069484.118)
Supplement: Supplemental Material [file supp_069484.118_Supplemental_Tables.docx]

**Supplementary tables:**

**Table 1: guide RNAs, ssDNA oligos, morpholinos**

| **sgRNA** | **Sequence 5’-3’** |
| --- | --- |
| sgRNA *cyrano^ΔCR^* 1 | GTATAGTAGTTCCTATCATA |
| sgRNA *cyrano^ΔCR^* 2 | CATCATTTAATGGAAGACAT |
| sgRNA *cyrano^ΔTSS^* 1 | GGTCCCGTGTGCTGCTACTG |
| sgRNA *cyrano^ΔTSS^* 2 | GTTGTTCGGGCCAGGCTCTG |
| sgRNA lnc-*sox4a^ΔTSS^* 1 | GCATGATATCGGACAAGGGG |
| sgRNA lnc-*sox4a^ΔTSS^* 2 | GACATCCTGACGTAGGTAAA |
| sgRNA lnc-*sox4a^Δ3’exon^* 1 | GTAAGATCATGGGTCCATAC |
| sgRNA lnc-*sox4a^Δ3’exon^* 2 | GTGAAGCGACTGACGCTGGT |
| sgRNA lnc-*pou2af1^ΔTSS^* 1 | GTGTGCGACCCGGCAGTGAA |
| sgRNA lnc-*pou2af1^ΔTSS^* 2 | GAGCGGAATGCGCAGAAAGT |
| sgRNA *malat1^polyA^* | GGTGAGGCGCTATGGAAGGC |
|  | |
| **ssDNA oligos** | **Sequence 5’-3’** |
| ssDNA *malat1^polyA^* | AACATTGTGCGTCACGACGGGGTGAGGCGCACTTGTTTATTGCAGCTTAAATGGTTACAAATAAAGCAATAGCATCACAAATTTCACAAATAAAGATTTTTTTCACTGCATTCTAGTTGTGGTTTGTCCAAACTCATCAATGTATCTTATCATGTCTGTTATGGAAGGCAGGGAGGCTTCGTTGATCTG |
|  | |
| **Morpholinos** | **Sequence 5’-3’** |
| *xrcc4* MO | CACTACTGCTGCGACACCTCATTCC |
| *xrcc5* MO | CCTTATTCTCAGCAAACACCTGCAT |

**Table 2: Genotyping primers**

| **Primer names** | **Sequence 5’-3’** |
| --- | --- |
| *cyrano^ΔCR^* Fwd | ACCACAGCTCAGAGCCACACTTGGA |
| *cyrano^ΔCR^* Rev | CTGGCACTACAAATCCCGCCACCCT |
| *cyrano^ΔTSS^* Fwd | CAATTTGTTCTCTTCAATTTTACCCTCGTCC |
| *cyrano^ΔTSS^* Rev | TGGAGTGAAGAGTAATTTTCAACAAATTTG |
| lnc*-sox4a^ΔTSS^* Fwd | TCCCACAAACTGTTTCCAACC |
| lnc*-sox4a^ΔTSS^* Rev | TCAGGACTTGGACCCAATGG |
| lnc-*sox4a^Δ3’exon^* Fwd | TAAGGAGCACAAATGTCTTAATACCTCAGG |
| lnc-*sox4a^Δ3’exon^* Rev | GTTTTCTCTATATGCCGACTGTTTTGATCC |
| lnc*-pou2af1^ΔTSS^* Fwd | GGGCTACAAATATCAGTGAAACTG |
| lnc*-pou2af1^ΔTSS^* Rev | CATTTACCAACGCTCTAGCTG |
| *malat1^polyA^* flanking Fwd | GTGTGGTATGTTGTGTCAAG |
| *malat1^polyA^* flanking Rev | CCGCCATTTTGTAAATTCTTTCTAGCGTCGAG |
| *malat1^polyA^* insert Fwd | TCACTGCATTCTAGTTGTGGTTTGTCC |
| *malat1^polyA^* insert Rev | GCTTGTATTTTATCTTCGTCACGCTTGC |

**Table 3: qPCR primers and primers to amplify RNA blot probes**

| **qPCR primers** | **Sequence 5’-3’** | |
| --- | --- | --- |
| qPCR *cyrano* Fwd | AAACCTTTCTAGCGGGGTGC | |
| qPCR *cyrano* Rev | TGATCCAAGTGTGGCTCTGAG | |
| qPCR lnc-*sox4a* Fwd | CATCACTCACAGTTCAGCTCTCC | |
| qPCR lnc-*sox4a* Rev | GAACACGACTATCCTCCACACTC | |
| qPCR lnc-*pou2af1* Fwd | CCTAAATCTCTAGGTATCGTTCAACTGGG | |
| qPCR lnc-*pou2af1* Rev | GCAAATGATATGAGTAAGCATTGCGTGAC | |
| qPCR *malat1* Fwd | CGTCACCTGAATGCAAGTGC | |
| qPCR *malat1* Rev | CCCCAAAATCCCCAGTCGAA | |
| qPCR *eef1α1* Fwd | CAGCATTATCCAGTCCTTAAGTAGAGTGC | |
| qPCR *eef1α1* Rev | GCGTCATCAAGAGCGTTGAGAAG | |
|  | | |
| **RNA blot primers** | **Sequence 5’-3’** | |
| *cyrano* Fwd | GGCTCAGTAGCTTAGAATACGCAGG | |
| *cyrano* Rev | TAATACGACTCACTATAGGGCTTCATGAGGATAATGAGTCATCAG | |
| *malat1* Fwd | GGGTGTAAAGCGCCGCTACC | |
| *malat1* Rev | GCGGTAATACGACTCACTATAGGGCTTGCATTCAGGTGACGTGATCC | |
|  | | |
| **5’ RACE primers** | | **Sequence 5’-3’** |
| 5’ RACE *cyrano* | | AACAATATGACCAGTCGATGGCACC |
| 5’ RACE *cyrano* nested | | ACACAAGAAGAGTTTGTGGGGGAGT |
| 5’ RACE lnc-*sox4a* | | ATCAGCCTTAGGTTACAGGAAGAGAGCC |
| 5’ RACE lnc-*sox4a* nested 1 | | CCAGAACACGACTATCCTCCACACTCGG |
| 5’ RACE lnc-*sox4a* nested 2 | | TTCCCACGCTGAAGGCTGATACTGAGAG |
| 5’ RACE lnc-*pou2af1* | | CATGGCTGCTATCATATGCCACGCCCACTAC |
| 5’ RACE lnc-*pou2af1* nested | | GTGTTTCTAAGGGAGACTCCAACCACCAAGTCC |
